# From Molecules to Multidisciplinary Teams: A Contemporary View of Heart Failure Care

**DOI:** 10.3390/jcm15135080

**Published:** 2026-06-29

**Authors:** Francesco Maria Animati, Dario Longo, Luigi Cappannoli, Cristina Aurigemma

**Affiliations:** 1 Dipartimento di Scienze Cardiovascolari e Pneumologiche, Università Cattolica del Sacro Cuore, 00168 Rome, Italy; francescoanimati@gmail.com (F.M.A.); dario.longo73@gmail.com (D.L.); 2 Dipartimento di Scienze Cardiovascolari—CUORE, Fondazione Policlinico Universitario A. Gemelli IRCCS, 00168 Rome, Italy; aurigemma.cristina@gmail.com

## 1. Introduction

With the closing of this Special Issue of the *Journal of Clinical Medicine*, the studies collected here seem to converge toward a common message. For two decades, the heart failure (HF) community has organized its hopes around the next drug and the next device. At a closer look, however, a quieter pattern emerges: across the HF spectrum, one factor that often determines whether therapeutic innovation translates into clinical benefit is not the intervention alone, but the system that delivers it.

The contributions gathered in this Special Issue span the entire continuum of HF care, from chronic HF management and acute decompensation to cardiogenic shock, remote monitoring, structural interventions, emerging therapeutic strategies, and precision medicine. Although these topics may appear heterogeneous, they collectively reinforce a common concept: innovation achieves its greatest impact when it is embedded within multidisciplinary systems of care, highlighting the importance of structured care pathways, multidisciplinary expertise, technological innovation, and patient-centered management.

This Editorial aims to provide an overview of the contributions published in this Special Issue and to interpret them through a unifying perspective: the growing role of team-guided management across the HF continuum. The most recent evidence in HF increasingly suggests that the decisive—and still underappreciated—therapeutic agent is teamwork. In the modern era, that team is expanding in two directions at once: outward, to include the patient as an active partner in a genuine therapeutic alliance, and technologically, to incorporate remote monitoring platforms and, prospectively, artificial intelligence-supported tools into clinical pathways. From this perspective, rather than focusing exclusively on new drugs or devices, teamwork itself may be regarded as a fifth pillar of contemporary HF care, able to organize multidisciplinary systems and to translate innovations into better patient outcomes.

## 2. Chronic Heart Failure

In chronic HF, both with preserved ejection fraction (HFpEF) and reduced ejection fraction (HFrEF), improving prognosis has become an increasingly complex challenge. Although guideline-directed medical therapy (GDMT) has profoundly transformed outcomes [1], contemporary HF management extends far beyond the prescription of evidence-based drugs. The clinical course of these patients is shaped by a dynamic interplay between congestion, renal dysfunction, frailty, multimorbidity, biological heterogeneity, and disease-specific phenotypes, all of which influence therapeutic decisions and frequently limit the implementation of optimal treatment. As a consequence, the central challenge is no longer simply identifying the most effective therapies, but ensuring that they are continuously adapted to the patient’s evolving clinical condition through a comprehensive and multidisciplinary evaluation.

This broader perspective is well reflected in the contributions collected in this Special Issue, which collectively illustrate that optimizing HF therapy requires the integration of multiple and often interconnected dimensions of the disease. Torres et al. [2] highlight how the implementation of quality-of-care measures in hospitalized HF patients remains a critical determinant of outcome, reinforcing the concept that translating evidence into structured clinical pathways and organized follow-up programs is as important as the therapies themselves. Within these pathways, Campos-Sáenz de Santamaría et al. [3] highlight the growing importance of cardiorenal interactions, emphasizing how the integration of bedside imaging and clinical evaluation may support individualized therapeutic decisions. The complexity of this process is further amplified in older and frail patients, where Montalto et al. [4] show that frailty, multimorbidity, malnutrition, polypharmacy, and palliative care needs frequently reshape therapeutic priorities and require integrated care networks extending beyond traditional cardiology. This multidimensional view of HF is reinforced by the growing recognition that cardiovascular diseases frequently extend beyond the heart itself. Birtolo et al. [5] explore the heart–gut axis, illustrating how cardiovascular disorders may present with severe extra-cardiac manifestations that require a broader diagnostic perspective and careful multidisciplinary evaluation. At the biological level, Ahmed et al. [6] underline the growing importance of inflammatory mechanisms as potential therapeutic targets, supporting the transition toward more individualized treatment strategies. Likewise, Poledniczek et al. [7] highlight how specific HF phenotypes may involve complex systemic manifestations requiring dedicated disease-specific expertise and multidisciplinary management.

Taken together, these observations help explain why, particularly in HFrEF, the dominant challenge is no longer simply what to prescribe but whether evidence-based therapies can actually be delivered, continuously optimized, and adapted to the evolving clinical condition of the individual patient. Therapeutic inertia and chronic under-titration continue to leave many patients undertreated despite the availability of the “four pillars” therapy (a renin–angiotensin system inhibition—ideally an angiotensin receptor–neprilysin inhibitor—a beta-blocker, a mineralocorticoid receptor antagonist, and a sodium–glucose cotransporter-2 inhibitor), which has dramatically improved survival in HFrEF [1].

The STRONG-HF trial [8] provided robust evidence supporting this concept, testing not a new drug but an intensive strategy based on the rapid optimization of GDMT coupled with close and structured follow-up after an acute HF admission. The strategy reduced the 180-day risk of all-cause death or HF readmission and improved quality of life. This result is even more striking when one considers that it was achieved using the same therapies already established for HFrEF and HFpEF; tellingly, the proportion of patients receiving optimized therapy rose to 93.5% under high-intensity care versus 36% with usual care. Perhaps the most important lesson from STRONG-HF is that the active ingredient was not a novel pharmacological intervention, but a system capable of continuously reassessing the patient and adapting treatment accordingly.

It is precisely this body of evidence that underpins the strong (Class of Recommendation I, Level of Evidence A) ESC HF Guidelines recommendation for multidisciplinary HF management programs [1]. The team itself has become an evidence-based intervention, as worthy of deliberate implementation as any drug or device.

Taken together, STRONG-HF, the ESC Guidelines, and the contributions collected in this Special Issue support a broader paradigm. Rather than representing separate fields of investigation, these studies collectively illustrate the multiple dimensions that shape contemporary HF management. Organizational quality, cardiorenal interactions, aging and frailty, systemic manifestations, biological heterogeneity, and disease-specific phenotypes all influence therapeutic decision-making and frequently interact with one another. In this setting, the multidisciplinary team is not simply an organizational model but the clinical framework that allows this complexity to be interpreted and translated into individualized care, ultimately enabling the full benefits of modern HF therapies to reach the patient.

## 3. Acute Heart Failure and Cardiogenic Shock

Nowadays, it is increasingly clear that the management of acute heart failure (AHF) and cardiogenic shock (CS) depends not only on the availability of advanced therapies but also on the ability to continuously reassess patients and promptly adapt treatment strategies. Botti et al. [9] describe the dynamic evolution of CS, showing how progression toward higher SCAI stages and persistent hemodynamic and metabolic deterioration are closely associated with adverse outcomes. Their findings support the need for continuous monitoring, early recognition of deterioration, and timely escalation of therapy, thereby reinforcing the role of dedicated multidisciplinary shock teams capable of integrating reperfusion strategies, hemodynamic assessment, pharmacological support, and advanced mechanical circulatory support.

These principles are not confined to CS but extend across the entire spectrum of AHF. Cannata et al. [10] analyzed more than 227,000 consecutive AHF admissions and demonstrated that specialist multidisciplinary HF care was associated with greater use of guideline-directed therapies and improved short- and long-term outcomes across the entire ejection-fraction spectrum. No new drug or device explains this difference: the determinant is how and by whom the patient is managed.

These observations are strongly supported by the broader AHF and CS literature. For many years, no pharmacological or device-based therapy convincingly improved survival in CS, and it became increasingly evident that only high-volume centers with well-structured shock teams could effectively address the substantial mortality associated with these syndromes [11].

The DanGer Shock trial [12] provided the first randomized evidence that mechanical circulatory support could improve survival in STEMI complicated by cardiogenic shock. However, this landmark result cannot be separated from the context in which it was achieved: experienced high-volume centers, carefully selected patients, and multidisciplinary teams able not only to implant the device but also to manage its complications and continuously adapt treatment strategies. Perhaps the most important lesson from DanGer Shock is that the benefit was generated not by the device alone, but by the system of care that delivered it.

In diseases where no single therapy uniformly changed prognosis, the multidisciplinary team becomes the platform on which the right intervention can be matched to the right patient at the right time.

## 4. The Patient, the Sensor, and the Algorithm

Much of heart failure prognosis is determined outside the clinic, through day-to-day decisions about symptoms, sodium and fluid intake, treatment adherence, and the timely recognition of clinical deterioration. In this evolving scenario, the contributions collected in this Special Issue suggest that the future of HF management will increasingly depend on the integration of innovative therapies and technologies within structured and multidisciplinary models of care.

This broader evolution is comprehensively reflected in the contribution by Tedeschi et al. [13] which outlines how the next generation of HF care will rely on the integration of optimized guideline-directed medical therapy, novel cardio–renal–metabolic therapies, advanced HF strategies, transcatheter structural interventions, and system-level approaches aimed at delivering individualized care across the HF spectrum. In particular, the authors highlight the growing role of emerging pharmacological options, such as finerenone, in reshaping the cardio–renal–metabolic continuum, while discussing new perspectives for advanced HF and structural heart disease. Rather than representing isolated innovations, these strategies are presented as complementary components of an increasingly personalized and multidisciplinary model of care, in which organizational pathways, technological advances, and disease-specific expertise work together to improve patient outcomes.

Within this broader vision of increasingly individualized and multidisciplinary care, Rdzanek et al. [14] provide an example of how emerging structural interventions may expand therapeutic opportunities for highly symptomatic patients with severe tricuspid regurgitation who are ineligible for conventional transcatheter or surgical approaches, further broadening the therapeutic armamentarium available for complex end-stage HF populations.

The multidisciplinary and technology-oriented perspective emerging from these contributions naturally extends to the growing body of evidence supporting remote monitoring strategies. By making clinical information continuously available and actionable, remote monitoring transforms the therapeutic alliance from an aspirational concept into a practical model of care, reframing the patient from a passive recipient into an active co-manager of the disease. [15].

Hemodynamic-guided care is perhaps the clearest example of this evolving paradigm. The CHAMPION [16] and MONITOR-HF [17] trials demonstrated that the continuous assessment of pulmonary artery pressure allows clinicians to anticipate congestion and prevent decompensation, emphasizing that the true value of technology lies in its integration within a responsive clinical team.

The same organizational principles also underpin non-invasive remote management. In TIM-HF2 [18], the benefit of telemedicine was closely linked to the presence of a structured telemedical center, trained nurses, and organized patient education, confirming that technology alone is insufficient without an appropriate organizational framework.

The future-oriented perspective proposed by Tedeschi et al. naturally extends to the growing role of artificial intelligence, which may support HF care from the earliest stages of diagnosis through treatment optimization. Studies such as the AI-enabled ECG investigations by Attia et al. [19] and the EAGLE trial [20], together with the broader diagnostic applications summarized by Xie et al. [21], demonstrate the potential of AI to identify disease earlier and more efficiently. Likewise, the AIM-POWER trial [22] illustrates how AI may facilitate the optimization of GDMT beyond the clinic by translating home-monitored data into actionable therapeutic recommendations. In many respects, this perspective brings us back to the central lesson of STRONG-HF: the key determinant is not the technology itself, but the continuous and coordinated optimization of care that it enables, aligning the patient, the clinical team, sensors, and algorithms toward a common therapeutic goal.

Read together, the contributions by Tedeschi and Rdzanek, together with the broader evidence from STRONG-HF, TIM-HF2, CHAMPION, MONITOR-HF, EAGLE, AIM-POWER, and the growing literature on precision medicine and emerging therapeutic strategies, tell a single story. In each case, the headline intervention—a device, a sensor, an algorithm, or a biologically informed approach—delivered its benefit because it was embedded within a coordinated system of care and, increasingly, within an alliance with an informed and engaged patient.

## 5. Conclusions

The contributions collected in this Special Issue provide a contemporary overview of heart failure across the continuum of care, capturing a pivotal transition in cardiovascular medicine from isolated therapeutic advances toward integrated, patient-centered, and phenotype-aware management. Across the studies presented here, heart failure emerges as a dynamic and multisystem syndrome, in which cardiorenal interactions, frailty and comorbidity burden, biological heterogeneity, inflammatory pathways, disease-specific phenotypes, structural heart disease, and acute haemodynamic instability are closely interconnected and collectively shape clinical decision-making and patient outcomes.

When integrated with the broader developments discussed above, including remote monitoring, artificial intelligence, precision medicine, and emerging therapeutic strategies, these insights suggest that future progress in heart failure will depend not only on the availability of new therapies, devices, or technologies, but also on the ability to organize them within coherent and responsive care pathways. In this setting, the multidisciplinary team is not merely an organizational model, but the clinical infrastructure through which disease complexity is interpreted, evidence is individualized, and innovation is translated into meaningful improvements in patient outcomes. 
